# Privacy Attitudes among Early Adopters of Emerging Health Technologies

**DOI:** 10.1371/journal.pone.0166389

**Published:** 2016-11-10

**Authors:** Cynthia Cheung, Matthew J. Bietz, Kevin Patrick, Cinnamon S. Bloss

**Affiliations:** 1 Center for Wireless and Population Health Systems, Calit2, University of California San Diego, La Jolla, California, United States of America; 2 Department of Informatics, Donald Bren School of Information and Computer Sciences, University of California Irvine, Irvine, California, United States of America; 3 Department of Family Medicine and Public Health, School of Medicine, University of California San Diego, La Jolla, California, United States of America; 4 Department of Psychiatry, School of Medicine, University of California San Diego, La Jolla, California, United States of America; Scripps Health, UNITED STATES

## Abstract

**Introduction:**

Advances in health technology such as genome sequencing and wearable sensors now allow for the collection of highly granular personal health data from individuals. It is unclear how people think about privacy in the context of these emerging health technologies. An open question is whether early adopters of these advances conceptualize privacy in different ways than non-early adopters.

**Purpose:**

This study sought to understand privacy attitudes of early adopters of emerging health technologies.

**Methods:**

Transcripts from in-depth, semi-structured interviews with early adopters of genome sequencing and health devices and apps were analyzed with a focus on participant attitudes and perceptions of privacy. Themes were extracted using inductive content analysis.

**Results:**

Although interviewees were willing to share personal data to support scientific advancements, they still expressed concerns, as well as uncertainty about who has access to their data, and for what purpose. In short, they were not dismissive of privacy risks. Key privacy-related findings are organized into four themes as follows: first, personal data privacy; second, control over personal information; third, concerns about discrimination; and fourth, contributing personal data to science.

**Conclusion:**

Early adopters of emerging health technologies appear to have more complex and nuanced conceptions of privacy than might be expected based on their adoption of personal health technologies and participation in open science. Early adopters also voiced uncertainty about the privacy implications of their decisions to use new technologies and share their data for research. Though not representative of the general public, studies of early adopters can provide important insights into evolving attitudes toward privacy in the context of emerging health technologies and personal health data research.

## Introduction

Rapid technological advances have enabled the development of personal genome sequencing, wearable health devices and apps, and other emerging health innovations. These innovations are becoming more widely disseminated to researchers and individuals alike. The ability to sequence and interpret individual genomes, or track and store continuous individual heart rate data, for example, has vastly expanded the personal health data ecosystem.

The enormous amounts of personal health information produced by these emerging health technologies can be powerfully revealing of the person behind the numbers [1]. For instance, these technologies can provide highly accurate information about an individual’s health status, physical location, activity levels, or other indicators of physical and social status [2]. As such, many have noted how these technologies challenge conceptualizations of individual privacy. Despite a growing recognition of the tangible benefits of emerging health technology, scholars question how people will think about privacy in the context of these health advances.

In the digital age, individuals may feel powerless to control the flow of their personal health data, and thus their privacy. For instance, while current marketing theory positions the consumer as a powerful decision maker who controls the entities that can access their data, recent research refutes this idea. Some have argued that consumers feel data mining is inevitable, and believe it would be useless to try and control who has access to their personal information [3]. Another body of literature suggests that if given a choice, individuals may be willing to trade their personal data, and by extension, their privacy, for certain benefits (e.g., enable location tracking on their phone for access to navigation services). It is unclear, however, whether individuals exhibiting these tradeoff behaviors are simply resigned to revealing personal data and are only trying to maximize their own benefit from data mining activities by third parties that will proceed either way [3]. Individuals may also be unaware of the granularity of their personal health data, its value, and the extent to which it can be combined with other sources of publicly available big data to recover even more personal information, such as specific identities.

Americans are also generally unaware of privacy law as it concerns their personal data [4], which is unsurprising, given that privacy policies are often written beyond the comprehension level of the average person [5–7]. For instance, the Genetic Information Nondiscrimination Act (GINA) protects individuals against discrimination based on their genomic data [8], but a recent study found that nearly 80% of the respondents sampled were unfamiliar with this legislation [9]. Moreover, current privacy laws encompass only personal information that is directly related to an identified or identifiable individual. In addition, no broad regulatory frameworks (e.g. HIPAA) exist to protect the information that can be derived from the compilation and cross-referencing of personal information with publicly available datasets [10].

One framework to better guide this discussion comes from renowned privacy scholar Alan Westin, who organized privacy beliefs into three categories reflecting different levels of privacy concern. These categories include:

᠅ Privacy fundamentalists: individuals who perceive their privacy to be at risk and actively resist further infringements upon their privacy (roughly 25% of the general public) [11];
᠅ Privacy pragmatists: individuals who are concerned about privacy but are willing to tolerate a certain amount of risk in exchange for personal or societal benefit (roughly 55% of the general public) [11]; and
᠅ Privacy unconcerned: individuals who are not particularly troubled by matters of privacy and do not actively consider the subject (roughly 20% of the general public) [11].

Although these definitions appear to be straightforward, the factors that influence an individual’s attitude toward privacy are likely complex and potentially nuanced. Indeed, privacy has been long been thought of as difficult to define [12] and has even been referred to as a “concept in disarray” [13]. Despite being an ill-defined construct, in the ever-expanding personal health data ecosystem, understanding conceptions of individual privacy has, perhaps arguably, never been more important. Along these lines, an open question is whether early adopters of emerging health technologies conceptualize privacy in different ways than non-early adopters. This study sought to understand attitudes toward privacy among early adopters of emerging health technologies.

## Methods

This project was reviewed by the Institutional Review Board (IRB) of the University of California, San Diego via the UC Reliance Registry (UC IRB Reliance #711) and was approved by the University of California, San Diego (UCSD) and the University of California, Irvine (UCI). Written consent was obtained for participants in the Personal Genome Project Perceptions Study and verbal consent was obtained for participants in the Health Data Exploration Project. These consenting procedures were approved by the IRB of UCSD, as were all other recruitment and study procedures.

Qualitative data from interviews with 18 individuals who participated in two prior studies were reanalyzed with a focus on themes related to individual privacy. These studies both focused on individuals’ willingness to collect personal health data derived from emerging health technologies and share those data with health researchers. Though the initial studies and interview protocols were broad in scope, privacy emerged as a significant issue that was discussed by all of the participants. As such, we have combined the data from both studies and performed an analysis to better understand privacy attitudes among early adopters.

### Study 1: Personal Genome Project Perceptions

The original intent of this study was to understand data sharing perceptions held by participants in the Personal Genome Project (PGP). The PGP is an ongoing endeavor to compile genomic information and other health data for the purposes of collaboration and increasing scientific knowledge about the human genome. Participants join the PGP specifically to donate their genome and health data to create an open, public data resource. Participants must complete a rigorous enrollment and informed consent process that requires passing an enrollment examination to demonstrate understanding of the study and the potential consequences of making personal genomic data public [14]. More information about the PGP can be found online at personalgenomes.org.

The Genomes, Environments, Traits (GET) conference is organized by the PGP to allow participants to gather and learn more about the project and related initiatives, and also to provide an opportunity to participate in additional research studies. At the GET 2014 conference we recruited a convenience sample of GET attendees to participate in semi-structured interviews at the conference expo. Individuals who expressed interest in participating were provided with a Study Information Sheet describing the study and given an opportunity to ask questions before being interviewed.

Interviews were conducted by one of the authors (MJB) who holds a Ph.D. in Information Science. Participants were told that the purpose of the interview was to understand how people think about and experience personal genetic testing. Interviews followed a semi-structured protocol that covered the respondent’s personal data collection (including self-tracking and genetic testing), history of participation in research studies, how the respondent makes decisions to participate, and specific experiences within the PGP project. All interviews lasted approximately 30 minutes. Our study sample consisted of 7 PGP participants (see Table 1 for descriptive information). The results of this study have not been previously published.

**Table 1:** Descriptive information of study participants.

Participant Characteristics		PGP (N = 7)	HDE* (N = 11)
Age	18–25	0	9.7%
	26–35	0	26.6%
	36–45	42.9% (N = 3)	23.0%
	46–55	0	21.6%
	56–65	42.9% (N = 3)	13.9%
	65+	14.3% (N = 1)	5.2%
Gender	Male	71.4% (N = 5)	34.5%
	Female	28.6% (N = 2)	65.2%
Race/Ethnicity	White/Caucasian	85.7% (N = 6)	79.3%
	Other	14.3% (N = 1)	20.7%
Education	Less than a 4-year college degree	14.3% (N = 1)	9.6%
	4-year college degree	42.9% (N = 3)	29.2%
	Postgraduate/professional degree	42.9% (N = 3)	61.1%
Marital Status	Yes	85.7% (N = 6)	65.6%
	No	14.3% (N = 1)	34.4%
Household Income	Less than $50,000	14.3% (N = 1)	23.7%
	$50,000-$75,000	14.3% (N = 1)	13.9%
	$75,000-$100,000	14.3% (N = 1)	15.2%
	$100,000-$150,000	57.1% (N = 4)	19.0%
	More than $150,000	0	28.2%
Employment Status	Currently employed	85.7% (N = 6)	85.3%
	Not currently employed	14.3% (N = 1)	14.7%

* Descriptive information of HDE survey participants who agreed to be interviewed. The actual interview participants reported on in this study were randomly selected from this pool.

### Study 2: The Health Data Exploration Project

The Health Data Exploration Project (HDE) involved surveys and interviews collected and conducted in summer and fall of 2013. This investigation was intended to deepen our understanding of the barriers to sharing personal health data for research. As part of the original study, a targeted convenience sample survey was conducted with 465 respondents. Participants were recruited through postings in social media, press releases, and related web pages. Survey respondents were asked if they were willing to participate in a follow-up interview, and a gender-balanced random sample was drawn from the subset of individuals who indicated interest. While participants were invited to participate by email or phone, all interviews were conducted over the phone. Participants were consented verbally according to the IRB-approved study protocol, and consent was documented by the interviewer. Although survey respondents in the original study included individual consumers, researchers, and companies/key informants, the follow-up interviews presented here were completed with individual consumers. Individuals drawn from this group (N = 11) were appropriate for our current study given that they were early adopters of personal health tracking technologies. The original study has been previously described [15], and additional information about the HDE Project can be found online at hdexplore.calit2.net.

Interviews were conducted by HDE project members who were faculty and post-doctoral scholars in public health and information science. Participants were told that the study was about self-tracking and other forms of personal data collection, data sharing, and willingness to share data with researchers. Interviews followed a semi-structured protocol that covered topics including individuals’ personal data collection practices (e.g. self-tracking) and their willingness to share personal data with researchers. Interviews lasted between 28 and 75 minutes, with the average duration being 45 minutes.

While privacy was identified as an emergent theme in the HDE interviews, this was not a primary focus of the original analysis. We have reanalyzed these interviews in the context of our current focus on attitudes toward individual privacy. Interviews have been anonymized, and as such, demographic information is no longer available for these interviewees. Demographic data for the sampling frame from which interviewees were recruited, however, are presented in Table 1.

### Participants as Early Adopters

We have characterized the participants in both of these prior studies as “early adopters” of personal health technologies. Early adopters are individuals who fall between one and two standard deviations from the mean in time needed to adopt an innovation [16]. They are described as individuals, organizations, or clusters of people who are risk-tolerant, well-educated, socially connected, science-minded and generally affluent [17, 18]. In the realm of health data technologies, early adopters are those who are more likely to engage with innovative self-tracking or medical devices [19] and share their personal health data with family members, medical providers, or researchers [18]. They are also closely watched by individuals in the early majority and are those who marketers most often target, based on their wide sphere of influence [19].

### Data analysis

Transcripts from these semi-structured interviews with participants in PGP and HDE were analyzed *de novo*. Our approach to analysis was adapted from grounded theory methods [20, 21]. We began with privacy as a guiding concept for our coding. Transcripts of interviews were open coded by two authors (CC and MJB) using the qualitative software program Dedoose 6.2.21. Through discussion and memoing, codes were refined and higher-level relationships (axial codes) and emergent themes were identified. These themes are discussed below.

## Results

Our sample of early adopters expressed more ‘pragmatic’ and ‘unconcerned’ type privacy beliefs, to reference the framework of Westin, than would be expected from the general public [11]. In spite of this, it is notable that participants were both highly aware of and actively considering ‘fundamentalist’ privacy concerns. Key findings have been organized into four themes: (1) personal data privacy is important but difficult to achieve; (2) respondents want control over their personal information; (3) discrimination based on personal data is a significant concern; and (4) respondents generally believe the benefits of contributing personal data to science outweigh the privacy risks. The quotations presented below are representative of participant responses and perspectives, and are meant to ground the themes that emerged across multiple participants in concrete examples from the interviews.

### Theme 1: Personal Data Privacy

Participants held a wide range of views about the privacy and security of their data. Among some, there was the sentiment that any shared personal health data would be “walled off from potential advertisers.” Other participants were more uncertain, bringing to light the issue of unclear privacy policies and the need to simplify user agreements:

“If someone can answer, "Here's where it's stored, here's how we use it," in simple ways, not this 30 page agreement. Very simply, "Here's where your data is stored. It's secure, safe," duh, duh, duh, and, "We use it in these kinds of reports, we use it for whatever." If they send it somewhere, someone should know that, right?” (HDE 01)

“I just saw a study the other day that said, "If you don't pay for the app, it's really bad”….Because your data's probably getting misused…. It was, basically, that the privacy policies they had in place weren't either that good, or weren't being enforced very well. It was worse for the free stuff as I recall. Something like that.” (HDE 09)

Several participants acknowledged the difficulty of protecting personal data in the digital age and expressed skepticism that participant data could truly be protected:

“I think that data can be protected, but it is hard…it can always be compromised….Especially if you have more than one researcher and you're sharing that data, then you never know what can happen.” (HDE 10)

Participants also had mixed feelings about their data being re-identified or somehow connected back to them:

“At last year’s PGP, they had set up a booth. They said, “Type in your gender, your zip code, and your birth date.” It says, “We’ve identified you from your voter records. Here, this is you.” I go, “Wow, that’s something.” Actually, I went into my PGP online file and I put XX for the last two digits of my zip code. Apparently, I am a little bit worried by it, but I’m not very worried by it… I don’t want to make it trivially easy for people to identify me…” (PGP 02)

“I really think that people don’t even… Nobody’s going to take the time. Who cares about my genetic information?” (PGP 04)

This prompted some to closely monitor their own data and disengage from the use of large data sharing platforms:

“I am concerned about privacy and who has access to my information. Also, Google shares that information as a result of a financial relationship they might have. For instance, I don't do Facebook anymore. I don't trust them not to share my information with companies that they acquire without telling me about it.” (HDE 05)

A small subset of individuals felt it was futile to try and control their privacy, and were resigned to being studied and known by research entities/marketers:

“Realistically, every time I go to the [Grocery Store], I'm in a study. Every time I go to [General Store], I'm in a study. Every time I go to Google, I'm in a study. I mean, right? Every time you go to Google, you're in a study. You don't think of it that way, but you are.” (HDE 11)

“…I hope [my data is] protected, but I'm also not naive and just assume it’s being used. It may be getting used in ways that I would not be happy about, but I'm not sure how much you can do. I mean we can do everything we can. HIPAA is the only real protector, I think, but even that has got its issues.” (HDE 09)

Across the interviewees, we noted an uncertainty regarding the ultimate fate of shared data. Even when participants expressed a good deal of recognition of the possible uses of their data, they felt unsure about the details of any particular data sharing policy. The wide spectrum of participant beliefs reveals that even among our sample of highly educated early adopters, data ownership and sharing policies are challenging to decipher.

### Theme 2: Control over personal information

For our informants, privacy concerns were intertwined with issues of ownership and access to personal information. All participants expressed the desire for control over their own data. Many participants believed they were full owners of the data they generated and expected to be in charge of releasing personal information at their discretion.

“I have no desire to have my weight information, or any of my health information hosted by a private company that I don't control access to. That's right. [This app] stores the data on my phone. It doesn't really go anywhere else… The fact that it doesn't store my information online was one of the reasons why I purchased it.” (HDE 05)

Some participants expressed a desire to control the use of their data even after it had been donated. For example, a data donor might want to rescind permission if circumstances change or their data is used inappropriately:

“I’m down with that… People can do whatever they want with our data… But what you’re trying to tell me is you’re now doing research that will put my name back on the data I gave you. In a way, you’re not just doing research on my data. You’re doing research on my data that will add data to my data that I didn’t give you for a reason.” (PGP 01)

Many participants also wanted to be able to access, compile and analyze their health data at their own convenience:

“I would like to have all of my health and medical records of any kind, including imaging data and test results, everything, under my own control. I would like to own my data and whenever I go to consult with a professional or a physician or a health care expert I'd like to be able to share that information with them and have them be privy to my entire health record history and I want to monitor it for problems and changes. That's my goal, to try and get complete control of my medical and clinical information.” (HDE 08)

One point of consensus among participants was the disapproval of monetizing or profiting from personal information. Alluding again to a kind of scientific altruism, participants felt that their data should be used for research with pure motives, not for the financial gain or profit of any individual or entity:

"Who owns the data? …My objection is not so much that my data will be aggregated and used for research. I'm OK with that. What I'm not OK with is for people purchasing to have access to my data so that they can then better target me or some other way monetizing my information. That's what I object to.” (HDE 05)

“What I don’t want is my information shared or monetized without my consent. And that’s one of the reasons I’m a little leery of using applications that put my name on any private entity’s website, because once you make contact…you get emails for the rest of your life….that’s the kind of relationship I’m not interested in.” (HDE 11)

Despite varying levels of concern about data usage, all participants desired increased control over their own data, both to have a better idea of how it was being disseminated and to analyze it for themselves. Participants tended to agree that if shared, their personal health data should only be used for altruistic purposes.

### Theme 3: Concerns about discrimination

Among the numerous themes that arose when discussing the risks and benefits of sharing personal health information, concern about discrimination was the most common risk identified across participants, especially in health insurance and employment. Participants cited both hypothetical and actual situations in which someone’s personal health data could be used in a discriminatory way:

“You know, you hear stories of how healthcare companies or health insurance companies are using the information to change the rates they’re charging people. I would have a negative reaction to that.” (HDE 02)

“Really, the only effect that PGP participation has had on me at all is a slight increase in anxiety over the fact that I might be identified…. I worry more about employment than insurance per se.” (PGP 01)

Importantly, however, none of our participants had experienced discrimination directly, instead referring to secondhand stories or “that kid that was in the news.” Notably, several participants mentioned that discrimination was unlikely to happen to them due to some perceived protective personal or situational factor:

“I really have nothing to fear…I teach out of college….I might be more wary if I was in my 20s and wanted to get a job, and I didn’t know what my [genetic] report showed. I have a somewhat elevated Alzheimer’s risk. Insurance companies can’t access the data right now. I have long-term care insurance. It’s possible that a long-term care insurance company wouldn’t give it to me, if [I didn’t already] have that. But I have it and they can’t break that contract as long as I pay.” (PGP 03)

Despite the very real possibility for unfair treatment due to genetic test results or health status, participants referenced age, employment status (i.e. being tenured), and lifestyle choices (i.e. low-risk behaviors) as factors that would be protective against discrimination in employment, insurance, or medical eligibility determinations.

### Theme 4: Contributing personal data to science

The majority of participants in our sample understood the act of sharing their personal health data to be a contribution to the growth of scientific research:

“I think the only way we’re going to understand our genomes is if a lot of us get sequenced and we’re willing to be studied. If we believe in the power of genetics, then we should be willing to do it.” (PGP 06)

“To be honest, I spent about 10 minutes looking at [my genetic report]. A lot of people here…they want to know about themselves. I’m not that interested. I don’t really care. I’m not saying you couldn’t convince me that there’s something of interest there, but I’m interested in helping science.” (PGP 01)

Many of our participants specifically expressed that they were willing to give up their anonymity and be subject to possible risks of sharing their personal health data for the benefit of the scientific enterprise:

“I think the main reason, we’re doing this for science. I think the good far outweighs the possible negative things that could happen. Don’t worry about those things.” (PGP 07)

“If you don’t take those risks and you don’t share information, you don’t have any benefits either. It’s like if you don’t try, you don’t get anything. If there’s going to be any progress, somebody’s got to have the balls to do something.” (PGP 04)

Participants reasoned that benefits for society outweighed the loss of privacy and the perceived intrusion into personal space. Other participants expressed this same scientific altruism, likening their contribution of personal health data to the idea of charitable giving:

“That’s what would motivate me, would be to have [my data] used for the good, for the good of the communities or for the good of whatever it is they’re researching. I guess when data can be used to help make a difference, and hopefully a positive difference, then that would be motivating to me to share it.” (HDE 10)

Numerous participants cited the desire to contribute to research and scientific knowledge as their main motivation for sharing their personal health data. Overall, the early adopters in our sample were generous with their data and were willing to tolerate the potential loss of privacy for scientific, and by extension, societal benefits.

## Discussion

A primary finding from this study is that early adopters of emerging health technologies appear to have more complex and nuanced attitudes toward privacy than might be expected based on their early adopter status and participation in open science. Traditional theories of early adopters have posited that they are only marginally concerned about significant negative outcomes [18], and thus would be expected to express more ‘privacy unconcerned’ beliefs. In contrast, our findings indicate that despite an enthusiasm for contributing personal data to science, early adopters maintain concerns similar to those of ‘privacy fundamentalists’ and ‘privacy pragmatists.’

It is not surprising that early adopters raised the issue of discrimination in the context of privacy and emerging health technologies; however, it is notable that participants in our study viewed discrimination as an issue they personally would be unlikely to encounter. Participants cited protective factors such as job tenure or existing insurance contracts that would insulate them against any conceivable discriminatory actions. Despite the fact that the health data collected from HDE participants are not covered by any broad regulatory framework (compared to the genomic data collected from PGP participants that have certain legal protections under GINA), these beliefs were present among interviewees from both cohorts. While there may be some validity to these claims that discrimination is a concern relevant for others, but less so for the individuals in our study, it is also possible that the perceived protective factors cited have more to do with rationalizing the decision to share personal data after the fact. This is consistent with findings from the literature positing that perceived risk and perceived benefit are often inversely related [22]. According to this paradigm, as perceived benefit increases, perceived risk is thought to decrease. Because the early adopters in this study perceived that their data were contributing significantly toward societal and/or scientific benefit, they may have been more willing to incur the associated risks of data donation. Interestingly, even though genetic data and, to a lesser extent other personal health data, are shared with biological relatives, participants did not cite risks to family members as being of concern in the context of discrimination. This suggests that this paradigm of risk and benefit may strongly influence participant beliefs and actions.

Early adopters also voiced uncertainty about the privacy implications of their decisions to use new technologies and share their data for research. This is notable given that our informants are highly educated and informed participants. PGP members have gone through one of the world’s most stringent consent processes [23] designed to ensure thorough awareness of potential worst case scenarios, but have continued to enroll and actively participate in ancillary studies. Similarly, participants in the HDE study were remarkably knowledgeable about self-tracking and emerging health technologies. Despite this, however, interviewees noted uncertainty in their understanding of data sharing policies and terms of use, and were nearly unanimous in their desire to have control over their own data.

Privacy protections have historically been maintained by de-identifying sensitive health data and obtaining informed consent from research participants. New research contends that these approaches are no longer valid, as researchers have demonstrated the ability to re-identify individual participants from aggregate data [24]. The de-identification of personal health information is now considered a temporary remedy [25] applied to an ever-evolving problem, one that poses a continual privacy risk to research participants [26]. Likewise, recent literature has deemed the informed consent process an ineffective formality, as participants often “do not have the time nor the expertise to decipher what is actually being said” or read [27]. Even when a privacy policy does explicitly state intended uses of personal health information, consumers are often unaware of the nature and extent of data sharing to which they are consenting [15]. For instance, a recent study of privacy policies found that multiple health tracking apps share sensitive user information with third parties, largely unbeknownst to consumers [24]. An open question is if participants actually understood the extent to which their data is being manipulated and shared, whether they would consent to such uses. Notably, a 2007 public opinion poll found that nearly 60% of individuals believe that health information privacy is not sufficiently protected, and over 30% of those who declined to participate cited concern about data confidentiality as the reason for not participating [28]. Clearly there is a need to earn and maintain public trust, and our data suggests this is no less of an issue when it comes to early adopters.

Though not representative of the general public, studies of early adopters can provide important insights into evolving conceptions of privacy in the context of emerging health technologies and personal health data research. Early adopters may be more science-minded and risk-tolerant than the early and late majority, but even they are not impervious to doubt about their decisions to use emerging health technologies, share the personal data that are collected, and participate in open science. Early adopters have provided an important glimpse into how emerging technologies challenge and shape individual attitudes toward privacy. Going forward, given their position as pioneers of culture change, these individuals will also serve as a critical resource as we consider how to best meet the privacy needs of individuals in the context of emerging health technologies and personal health data research.
